# Sonic Hedgehog—‘Jack-of-All-Trades’ in Neural Circuit Formation

**DOI:** 10.3390/jdb5010002

**Published:** 2017-02-08

**Authors:** Nikole R. Zuñiga, Esther T. Stoeckli

**Affiliations:** Department of Molecular Life Sciences and Neuroscience Center Zurich, University of Zurich, Winterthurerstrasse 190, 8057 Zurich, Switzerland

**Keywords:** axon guidance, commissural axons, midline crossing, synaptogenesis

## Abstract

As reflected by the term morphogen, molecules such as Shh and Wnts were identified based on their role in early development when they instruct precursor cells to adopt a specific cell fate. Only much later were they implicated in neural circuit formation. Both in vitro and in vivo studies indicated that morphogens direct axons during their navigation through the developing nervous system. Today, the best understood role of Shh and Wnt in axon guidance is their effect on commissural axons in the spinal cord. Shh was shown to affect commissural axons both directly and indirectly via its effect on Wnt signaling. In fact, throughout neural circuit formation there is cross-talk and collaboration of Shh and Wnt signaling. Thus, although the focus of this review is on the role of Shh in neural circuit formation, a separation from Wnt signaling is not possible.

## 1. Introduction

The best known function of Shh (Sonic hedgehog) is its role as a morphogen in the developing spinal cord [1,2,3,4,5,6]. Cells in the developing neural tube adopt a different cell fate depending on their position along the dorsoventral axis. Each position is specified by opposing gradients of morphogens. In the dorsal spinal cord, neurons are mainly influenced by Bone Morphogenetic Proteins (BMPs) and members of the Wnt family, secreted by the roof plate [7,8,9,10]. In the ventral spinal cord, the floor plate is the source of the ventralizing morphogen Shh.

**Figure 1:**
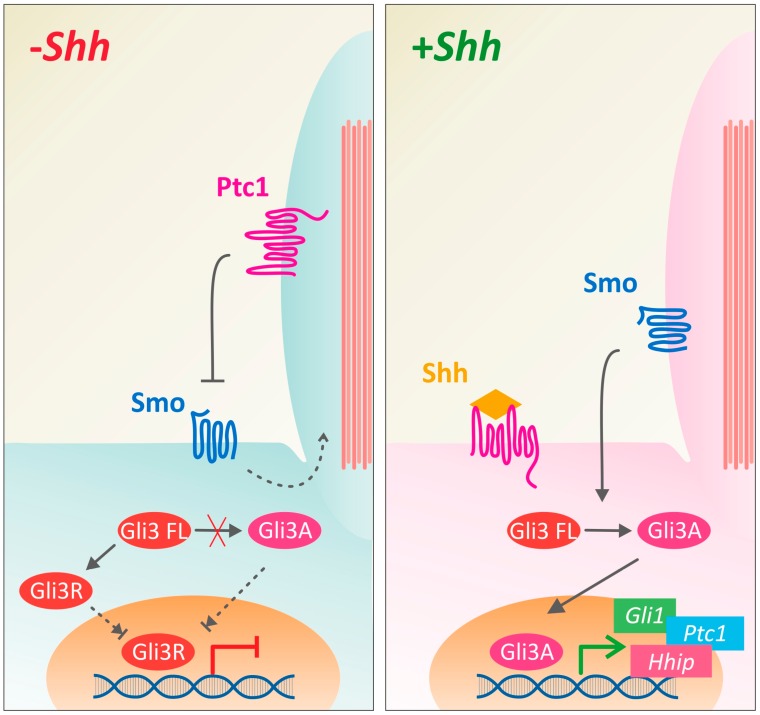
Canonical Shh signaling requires translocation of Smo into the primary cilium. Canonical Shh signaling depends on the de-repression of the G-protein-coupled receptor-like molecule Smoothend (Smo) by a mechanism involving the multiple-pass transmembrane receptor Patched1 (Ptc1). In the absence of Shh, Ptc1 represses Smo activity by preventing its translocation to the cilium. Shh binding to Ptc1 relieves this repression, allowing Smo to translocate to the primary cilium, where it activates intracellular signaling pathways. This activation of Smo culminates in transcriptional activity, via its regulation of Gli transcription factors that can activate or repress Shh target genes. Gli1 is always an activator of gene expression, whereas Gli2 and Gli3 (Gli3FL) need to be processed from a repressor (Gli3R) to an activator form (Gli3A) in a Smo-dependent manner.

Shh signaling affects cell differentiation by binding to the surface receptor Patched1 (Ptc1). In the absence of Shh, Ptc1 represses Shh signaling by keeping Smoothened (Smo) out of the primary cilium. Upon Shh binding, Smo translocates to the cilium, allowing for the processing of Gli from a repressor to an activator form, which in turn regulates the transcriptional program of the cell (Figure 1). Canonical Shh and Wnt signaling pathways converge in this process, as Gli expression and activation is also affected by Wnt signaling [11]. Thus, Shh and Wnt are important for the differentiation of neurons early on.

The role of Shh in axon guidance was identified much later than its function in cell differentiation and tissue patterning [12]. Initially, Shh was shown to inhibit the axon growth of retinal ganglion cells [13]. Later, Shh was implicated in commissural axon guidance in the spinal cord [14,15,16,17,18]. This role was extended to other populations of neurons [19,20,21,22,23].

## 2. Shh in Commissural Axon Guidance

Commissural neurons in the dorsal spinal cord have been used as a model to study molecular mechanisms of axon guidance mainly due to their accessibility to experimental manipulations and their stereotypic trajectory [24,25,26,27]. The dI1 subpopulation of commissural neurons [28] extends their axons towards the floor plate, which forms the ventral midline of the developing spinal cord. Axons cross the midline and turn rostrally towards the brain [29] along the contralateral floor-plate border. Axons navigate through the developing neural tissue based on the interaction of receptors expressed on the growth cone surface with guidance molecules in the environment [30]. Guidance cues can be classified into four different groups: long- and short-range cues, which can either be attractive or repulsive. By definition, long-range cues are released from a source and either attract growing axons towards the source (long-range attractants) or repel them from the source (long-range repellents). Due to their diffusible nature, long-range guidance cues outline the overall direction towards the target of the extending axons but they cannot specify the actual pathway. This is done by the integration of signals derived from the interaction between short-range guidance cues and receptors on the growth cone surface. Attractive short-range guidance cues pave the way to the target, whereas repulsive short-range cues inhibit axon growth.

For dI1 commissural axons, both long- and short-range guidance cues have been identified [24]. Axons are directed to the ventral spinal cord by the long-range repellents BMP7 and Draxin derived from the roof plate, the dorsal midline [31,32,33]. At the same time, they are attracted by long-range attractants derived from the floor plate. The major chemoattractant is Netrin1 [34,35]. Netrin1 is sufficient to attract pre-crossing axons toward their intermediate target, the floor plate, as shown recently in mice that express no Netrin1 at all [36,37]. The originally analyzed mice were hypomorphic for Netrin1, as they were generated by insertion of a marker into the *Netrin* gene (gene trap line) rather than its deletion resulting in reduced but still detectable Netrin protein expression [35]. However, the role of Shh as a long-range attractant for pre-crossing axons was only identified thanks to the fact that the hypomorphic Netrin mice were analyzed before the full knockout was available. In the hypomorphic *Netrin* mouse line, some axons still extended toward the floor plate, prompting the question of whether the residual levels of Netrin protein were responsible for their navigation, or whether additional long-range attractants would be released from the floor plate. Shh appeared to be a good candidate due to its strong expression in the floor plate during the time of commissural axon navigation.

### 2.1. Shh Is a Chemoattractant for Pre-Crossing Commissural Axons

**Figure 2:**
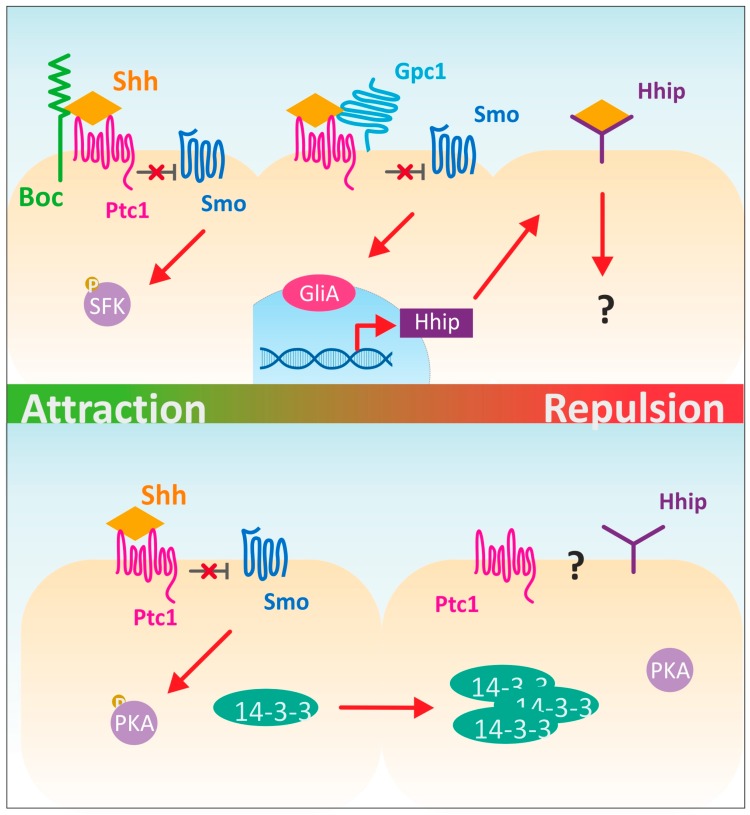
Non-canonical Shh signaling in commissural axon guidance. In vivo and in vitro studies have implicated Shh in commissural axon guidance. Shh is attractive for pre-crossing but repulsive for post-crossing commissural axons. On pre-crossing commissural axons Shh binds to Boc (Brother of Cdo) and Ptc1 (Patched1), thus de-repressing Smo (Smoothened). However, in contrast to canonical signaling (see Figure 1), binding of Shh results in the phosphorylation of Src family kinases (SFKs). Shh itself induces expression of Hhip (Hedgehog-interacting protein), its receptor for post-crossing axon navigation, by binding to Glypican1 (Gpc1) and Ptc1. The de-repression of Smo results in transcription of Hhip via the canonical signaling pathway. How Shh binding to Hhip on post-crossing commissural axons mediates repulsion is not yet known. In vitro (lower panel), protein 14-3-3 levels have been linked to the transition from attraction to repulsion. Pre-crossing axons initially express low levels of protein 14-3-3 (left). High levels of 14-3-3 were required for the repulsive response to Shh (right). It is unknown how 14-3-3 is linked to the precise switch from attraction to repulsion. Protein 14-3-3 can reduce the activity of PKA (Protein Kinase A), but no changes in the levels of cAMP were found between pre- and post-crossing axons. Furthermore, it is unknown if (and which) surface receptors could be linked to the function of protein 14-3-3.

Indeed, the analysis of Shh in a hypomorphic Netrin background resulted in the characterization of Shh as an additional long-range chemoattractant for dI1 axons [14]. The axons were attracted by Shh binding to its well-known receptor Ptc1 and the immunoglobulin superfamily member Boc (Brother of Cdo; Figure 2 [38]). Interestingly, the attractive effect of Shh on pre-crossing commissural axons was not mediated by canonical Shh signaling [16]. Axons were still attracted to Shh in the presence of transcriptional inhibitors. Instead, Shh was found to activate Src family kinases in dI1 neurons. In the presence of a Shh gradient, Src and Fyn are rapidly phosphorylated and distributed asymmetrically in the growth cone. Since Src family kinases can regulate cytoskeletal dynamics [39,40], the polarized distribution of phosphorylated Src and Fyn affects growth cone turning locally without affecting transcription.

### 2.2. Shh Is a Repellent for Post-Crossing Commissural Axons

Interestingly, in vivo studies in chicken embryos indicated a role of Shh in the guidance of post-crossing axons [15]. Because chicken embryos are readily accessible for manipulations in vivo in a precisely temporally controlled manner, Shh’s role on pre- and post-crossing axons can be analyzed separately [25,26,27]. Silencing Shh after its role in cell differentiation and its effect on pre-crossing axons indicated that Shh was a repellent for post-crossing commissural axons [15,18]. Initially, these results seemed to be at odds with the finding that Shh can attract pre-crossing commissural axons. However, the difference between the responses of pre- versus post-crossing commissural axons was shown to be the level of 14-3-3 protein expression [41] and the expression of Hhip (Hedgehog-interacting protein) as a Shh receptor [15,18]. In vitro experiments with rat commissural neurons exposed to a Shh gradient indicated a requirement of increasing levels of 14-3-3 proteins to explain the observed repulsion of axons by high Shh levels [41].

In vivo experiments in chicken embryos indicated that Shh itself triggered the expression of Hhip, its receptor mediating the repulsive response, by binding to Glypican1 expressed on pre-crossing axons [18]. As Glypican1 is not a transmembrane protein but rather a glycosylphosphatidyl-inositol anchored proteoglycan, it is unclear how Shh binding to Glypican1 triggers a change in transcription (Figure 2). The most likely explanation is that Glypican1 interacts with another molecule in the plane of the growth cone membrane. Whether this molecule is one of the known Shh-binding molecules Ptc1 or Boc remains to be shown. The induction of Hhip expression requires canonical Shh signaling [18]. Additional experiments will be required to identify the regulatory mechanisms in more detail and to show if and how the induction of Hhip and the increase in protein 14-3-3 levels are linked. The analysis of Hhip knockout mice did not indicate a requirement for Hhip in post-crossing commissural axons [41]. However, genetic redundancy can obscure the role of a particular protein in axon guidance, as has been shown in other cases. For instance, a role of Neuropilin2 in post-crossing commissural axon guidance was identified in mice, which could explain why post-crossing axons still turn rostrally in *Hhip* knockout mice [41,42].

At present it is unknown how Hhip transmits the Shh signal that results in repulsive axon guidance. Although Hhip is a type I transmembrane protein that can bind directly to all vertebrate hedgehog proteins [43], it does not contain an intracellular domain. Thus, it is unclear how Hhip would affect intracellular signaling. The most straightforward explanation would be that Hhip could mediate repulsive axon guidance by acting as a Shh-binding unit in a receptor complex containing an unidentified signal-transducing component. Alternatively, Hhip might modulate Shh signaling by sequestering it away from other receptors [44]. Consistent with this idea, Hhip has been reported as a negative regulator of Shh signaling [43], by competing with other Hedgehog receptors for Shh binding, including Cdo [45] and possibly Ptc [46].

### 2.3. Shh Collaborates with Wnts in Post-Crossing Commissural Axon Guidance

In addition to its direct effect on post-crossing commissural axons, Shh has an indirect effect [47]. Shh was shown to induce the expression of Sfrp1 (Secreted frizzled-related protein1), an endogenous antagonist of Wnt signaling, in an anterior^low^ to posterior^high^ gradient in the floor plate of chicken embryos in vivo. According to its expression pattern, Sfrp1 was shown to form a Wnt activity gradient along the anteroposterior axis of the developing neural tube. In mice, Wnt4 was shown to be expressed in an anterior^high^ to posterior^low^ gradient in the floor plate [48]. No such Wnt expression gradient was found in chicks, but the attractive effect of Wnt5a and Wnt7a due to the activity gradient shaped by Sfrp1 confirmed the role of attractive Wnt signaling on post-crossing commissural axons [47,48]. More detailed analyses of Wnt signaling in commissural axons indicated a complex network of components and receptors originally assigned to either the canonical or the planar cell polarity pathway of Wnt signaling [49,50,51]. Based on these studies, it is clear that the traditional segregation of Wnt signaling into distinct pathways is not valid for axon guidance, a conclusion also drawn for Wnt signaling in other contexts [52,53,54,55].

In addition to the indirect effect of Shh on Wnt activity, the two pathways also converge on Gli activation. In vertebrates, three Gli transcription factors (Gli1-Gli3) have been described [56,57,58,59,60,61,62]. Gli2 and Gli3 both have transcriptional activator and repressor activities, whereas Gli1 acts only as a transcriptional activator (reviewed by Ruiz i Altaba et al., 2007 [63]). Each Gli family member responds differently to Shh [64]. In the absence of Shh, Gli2 is fully degraded, while Gli3 is converted to a repressor of transcription (Gli3R). When Shh is present, Gli1 expression is induced and proteolysis of Gli2 and Gli3 is inhibited, allowing Gli activator forms to accumulate (Figure 1). In turn, in a feedback loop, Gli transcriptional activators then induce the expression of Shh target genes [65,66,67].

Shh and Wnt signaling cooperate via regulation of Gli3 [68]. Gli3R, the transcriptional repressor form of Gli3, is generated in the absence of Shh. Gli3R was shown to interact directly with β-Catenin and thus influence its role in Tcf3/4-mediated control of transcription, although the precise mechanism is currently unknown [69].

In addition to its direct effect on axon guidance and its indirect effect as a modulator of the axon guidance activity of Wnts, Shh was also suggested to regulate growth cone responsiveness via local intracellular mechanisms. Shh was shown to influence levels of cyclic nucleotides, which are important for signaling in response to guidance cues [13,70]. The ratio of cAMP to cGMP determines attractive versus repulsive axonal responses [71]. High levels of cAMP and low levels of cGMP favor attraction, whereas the opposite ratio of cAMP to cGMP favors repulsion. Since Shh can influence cyclic nucleotide levels by reducing the activity of protein kinase A (PKA), it may influence the responsiveness of axons to additional guidance cues, such as semaphorins [13,17].

## 3. The Role of Shh in Other Neuronal Populations

In contrast to the role of Shh in commissural axon guidance in the spinal cord, much less is known about the effect of Shh on other populations of neurons. Shh was also shown to be repulsive for serotonergic neurons forming the raphespinal tract in a Ptc1- and Smo-dependent manner [23] and for axons of enteric neurons in a Gas1-dependent manner [21].

Other effects of Shh were explained by an effect on axon growth rather than guidance. For instance, Shh was shown to inhibit the growth of retinal ganglion cell axons [13]. This effect was shown to involve intracellular levels of cAMP. A more recent study revisiting the role of Shh on retinal ganglion cell axons concluded that the effect was mediated by protein kinase Cα and integrin-linked kinase (ILK) [72].

In an in vitro model, Shh was found to positively affect regenerative neurite growth [73]. Shh added to the medium enhanced neurite lengths of cortical neurons exposed to reactive astrocytes. Similar to its effect on pre-crossing commissural axons [16], Shh was concluded to activate Src family kinases [73]. This is in contrast to a report on the effect of Shh on hippocampal neurons [74]. Hippocampal axon elongation was found to be stimulated by Shh in an Smo- and Gli1-dependent manner. Interestingly, Shh did not act directly on the axon but rather via the somatodendritic compartment, where the Shh-mediated Gli1 activation induced higher intracellular Profilin activity that induced axon elongation.

## 4. Shh at the Synapse

Upon reaching their appropriate target cell, axons initiate and stabilize contacts, by remodeling the growth cone into synaptic boutons and assembling pre-synaptic vesicle release machinery. Similarly, the post-synaptic cell must recruit receptors for neurotransmitters. Recent studies have demonstrated contributions of Wnts to synapse formation, plasticity, and maintenance in both vertebrates and invertebrates (reviewed by Salinas and Zou, 2008 [75]; Budnik and Salinas, 2011 [76]). Wnt7a was demonstrated to promote synapse assembly via its effector Dishevelled-1 (Dvl1) [77,78]. In pre-synaptic hippocampal neurons, this activity of Wnt7a was mediated by the receptor Frizzled5 (Fzd5) [79]. The expression of Dvl1 has been demonstrated to increase the stability of microtubules through a signaling pathway that inhibits GSK3β but does not require transcription [80]. Inhibition of GSK3β reduces phosphorylation of the microtubule-associated protein MAP1B, thus linking Wnt signaling to the regulation of microtubule stability and growth cone remodeling [81,82].

Wnt5a was demonstrated to stimulate post-synaptic assembly [83], suggesting that different Wnts might regulate synapse formation by promoting protein assembly in either pre- or post-synaptic cells. In the post-synaptic cell, Wnt5a does not act via GSK3β, but instead leads to clustering of the scaffold molecule PSD95.

Wnt signaling has also been demonstrated to promote synaptic plasticity. The expression and release of Wnt proteins is enhanced by depolarization [84,85]. High-frequency stimulation of hippocampal neurons increased the level of cell-surface Fzd5 and its localization to synapses, in a Wnt-dependent manner [79]. In addition, the remodeling of hippocampal circuits in response to an enriched environment was repressed when Wnt signaling was blocked [86]. Together, these studies link Wnt activity to synaptic remodeling in response to behavioral experience and activity.

In contrast to these roles of Wnts at synapses, there is currently only limited evidence that Shh signaling is directly involved in synaptogenesis or synaptic plasticity [87,88]. Shh and its receptors are appropriately expressed to support such activities. In the retina, Shh-N was shown to be transported along the optic nerve to the superior colliculus in vivo [89]. Similarly, Shh is distributed intracellularly in regulated secretory vesicles and on the extracellular surface of the soma, dendrites, and axons of rat retinal ganglion cells (RGCs). Shh secretion can be induced by depolarization of RGCs, suggesting a link between neuronal activity and Shh release [90]. Ptc and Smo were localized to dendrites and spines of mature rat hippocampal neurons, suggesting that Shh signaling may influence synaptic transmission [91]. Indeed, in a follow-up study, Shh was found to increase presynaptic terminal size in hippocampal neurons in vitro [92]. Furthermore, the addition of Shh to these cultured neurons increased the frequency, but not the amplitude, of mEPSCs (miniature excitatory postsynaptic currents) [93]. In an earlier study, Smo was also localized to the presynapse [94]. These findings match what was found in the only in vivo study done for the analysis of Shh function in synaptogenesis so far [88]. In cortical circuit formation, Shh and its receptor Boc were found in a layer-specific manner. The analysis of mouse brains lacking either Shh or Boc due to gene silencing by in utero electroporation revealed that connections between layer 2/3 axons and layer 5 dendrites require Boc in the presynaptic and Shh in the postsynaptic partner. These studies indicate that Shh most likely also acts during the last steps of neural circuit formation, synaptogenesis, and synaptic plasticity. However, many more studies will be required to elucidate the molecular mechanisms of Shh’s effect.

## 5. Conclusions

Taken together, in vitro and in vivo studies have clearly indicated the multifaceted role of Shh in development. In the nervous system, Shh and Wnt signaling are involved in all aspects of neural circuit formation, from cell differentiation to synaptic plasticity. During most of these steps, Shh and Wnt signaling cooperate and influence each other. The cross-talk occurs at different levels, from shaping extracellular activity gradients to the regulation of common downstream effectors. No other class of molecules has been identified that was shown to affect the development of neural circuits throughout the entire process from cell differentiation to the regulation of synaptic plasticity.
